# RNA‐guided Cas9 as an *in vivo* desired‐target mutator in maize

**DOI:** 10.1111/pbi.12739

**Published:** 2017-05-12

**Authors:** Chuxi Li, Changlin Liu, Xiantao Qi, Yongchun Wu, Xiaohong Fei, Long Mao, Beijiu Cheng, Xinhai Li, Chuanxiao Xie

**Affiliations:** ^1^ Institute of Crop Science Chinese Academy of Agricultural Sciences National Key Facility for Crop Gene Resources and Genetic Improvement Beijing China; ^2^ Anhui Agricultural University Hefei Anhui Province China; ^3^ AgGene Bio‐tech Seed Industry Group Shenzhen China

**Keywords:** genome editing, desired‐target mutator, sexual plant breeding

## Abstract

The RNA‐guided Cas9 system is a versatile tool for genome editing. Here, we established a RNA‐guided endonuclease (RGEN) system as an *in vivo* desired‐target mutator (DTM) in maize to reduce the linkage drag during breeding procedure, using the *LIGULELESS1* (*LG1*) locus as a proof‐of‐concept. Our system showed 51.5%–91.2% mutation frequency in T0 transgenic plants. We then crossed the T1 plants stably expressing DTM with six diverse recipient maize lines and found that 11.79%–28.71% of the plants tested were mutants induced by the DTM effect. Analysis of successive F2 plants indicated that the mutations induced by the DTM effect were largely heritable. Moreover, DTM‐generated hybrids had significantly smaller leaf angles that were reduced more than 50% when compared with that of the wild type. Planting experiments showed that DTM‐generated maize plants can be grown with significantly higher density and hence greater yield potential. Our work demonstrate that stably expressed RGEN could be implemented as an *in vivo* DTM to rapidly generate and spread desired mutations in maize through hybridization and subsequent backcrossing, and hence bypassing the linkage drag effect in convention introgression methodology. This proof‐of‐concept experiment can be a potentially much more efficient breeding strategy in crops employing the RNA‐guided Cas9 genome editing.

## Introduction

Programmable nucleases such as zinc‐finger nucleases (ZFNs), transcription activator‐like effector nucleases (TALENs) and RNA‐guided endonucleases (RGENs) have been developed as versatile genome‐editing tools to target genes in diverse species, including plants (Li *et al*., 2012; Porteus, 2009; Shan *et al*., 2013; Zhao *et al*., 2016), and constitute the basis for novel applications in crop plant genetic improvement. RGENs are rapidly superseding ZFNs and TALENs due to their ease of use (i.e. the flexible assembly of the Cas9 protein and customized guidance of the RNA component; Woo *et al*., 2015). Gene knockout was the earliest application of RNA‐guided Cas9, and RNA‐guided Cas9 has been applied to a number of crops to engineer targeted modifications (Ma *et al*., 2015; Miao *et al*., 2013; Shan *et al*., 2013, 2015; Wang *et al*., 2014; Xie and Yang, 2013; Zhang *et al*., 2014). Genome‐editing machinery such as RGEN induces targeted mutations in trans‐mode in target cells that can target its own and the recipients’ genome when the two genomes are sexually crossed. Based on this rationale, genome editing has been proposed to overcome linkage drag problems in breeding, irrespective of the transient or stable transformation of the genome‐editing machinery (Lin *et al*., 2014). The delivery of genome‐editing machinery into target cells is one of the key steps and strategies for generating heritable mutations in plants. Delivery of the machinery in genome‐editing protocols is accomplished by creating a target mutation via transient expression (Svitashev *et al*., 2015; Zhang *et al*., 2016) and RNP delivery (Woo *et al*., 2015) in any genotype background, without the regulatory concerns associated with genetically modified organisms (GMOs; Huang *et al*., 2016; Woo *et al*., 2015). However, plant regeneration from the protoplast, suspension cells and calluses is either impractical or highly dependent on the specific genotype and requires laborious and time‐consuming tissue culture steps in most major crop species, including maize, wheat, rice and soya bean (Birch, 1997). Thus, the stable transformation of a few easily transformed genotypes (Birch, 1997; Gupta and Ram, 2004) and the implementation of genome‐editing machinery in trans‐mode to generate intended mutations remain practical for most important species and major crop species.

One of the applications of the genome‐editing machinery is to reduce so‐called linkage drag during conventional breeding by introgression. Previous genomic analyses have indicated that the extensive linkage drag associated with genome segmentation covers nearly 25.6% of the assembled genome, limiting further improvement via genetic recombination at meiosis during breeding (Lin *et al*., 2014). Thus, attempts should be made to break the linkage of the target gene in breeding programs (Brown, 2002). However, such attempts are challenging and require large populations and laborious background genome selection to disrupt linkage because the recombination rate is quite low, with fewer than four crossovers per chromosome per meiosis being observed in *Zea mays* L. (Li *et al*., 2015). Therefore, stacking favourable genes without introgression breeding is of great value in plant breeding. Direct genome‐editing technology provides such an opportunity. However, an experimental proof‐of‐concept to validate this strategy is lacking.

Plant density is an important factor contributing to the grain yield per unit area. Historical grain yield data from the two most important maize producers (eight decades of data from the USA and four decades of data from China) showed that the obtained yield is not due to the kernel yield per plant but, rather, to increases in plant density (Ci *et al*., 2011; Duvick, 2005). Therefore, increasing plant density is an important method for continuing to increase the grain yield per unit area in modern maize (Brekke *et al*., 2011). Maize with upright leaves can be planted at higher densities and captures more light, which increases the grain yield (Lambert and Johnson, 1978). A study conducted in rice, another important cereal crop, also demonstrated this beneficial effect in practice (Sinclair and Sheehy, 1999). More erect upper leaves have been reported important during the development of historical hybrid maize varieties (Duvick, 2005; Hammer *et al*., 2009). Thus, erect upper leaves are of great value for maize breeding and in cultural practice and the genes responsible for such a phenotype has been a frequent target by genome editing in maize. The maize ligule and auricle are structures located at the hinge of the sheath and blade that allow the leaf to project at an angle from the culm. *Liguleless* mutants lack these structures (Becraft and Freeling, 1991; Fowler and Freeling, 1996; Moon *et al*., 2013), and field experiments on *liguleless* hybrids showed a potential for an increased grain yield (Lambert and Johnson, 1978). Four *liguleless* genes have been identified as the genetic basis for the upright architecture of maize leaves. Among these genes, the *LIGULELESS1 (LG1)* locus has been demonstrated to be strongly associated with the upper leaf angle (Tian *et al*., 2011). *LG1* encodes SQUAMOSA PROMOTER‐BINDING protein required for leaf ligule and auricle development (Moreno *et al*., 1997). Mutants harbouring a single recessive *LG1*‐null mutation (*liguleless1*,* lg1*) lack ligules and auricles and exhibit upright leaves (Becraft and Freeling, 1991; Johnston *et al*., 2014). Cell‐ and tissue‐specific genes that are differentially expressed along the proximal‐distal axis of the ligule region have been compared in detail between *lg1* and wild‐type plants. Many of these genes have been shown to interact with multiple hormonal signalling pathways (Johnston *et al*., 2014). Nevertheless, the mechanism by which *LG1* affects the formation of ligules and auricles and, consequently, the leaf angle has not yet been identified. An attempt to induce heritable, targeted mutagenesis of the *LG1* locus in maize was made using a designed I‐*Cre*I‐homing endonuclease, but a target site of the gene promoter was selected to demonstrate targeted mutagenesis in maize (Gao *et al*., 2010). A recent study (Svitashev *et al*., 2015) performed using the RNA‐guided Cas9 system in which *LG1* was examined targeted the same region to analyse and compare the obtained mutation frequency with a previous report (Gao *et al*., 2010). The obvious mutant phenotype of targeted editing on the regulatory region in the *LG1* promoter had not been reported (Gao *et al*., 2010; Svitashev *et al*., 2015).

Here, we report the establishment of an efficient RGEN system in maize by targeting the maize *LG1* locus. Engineered plants can easily spread the desired‐target mutations (DTM) through simply pollinating the elite recipient cultivars. The progeny with upright leaves can be grown with significant higher planting density. Such a system can be used to circumvent the traditional genetic linkage drag, significantly increasing breeding efficiency.

## Results

### Generation of transgenic maize lines harbouring *LG1*‐targeting RGEN

**Figure 1:**
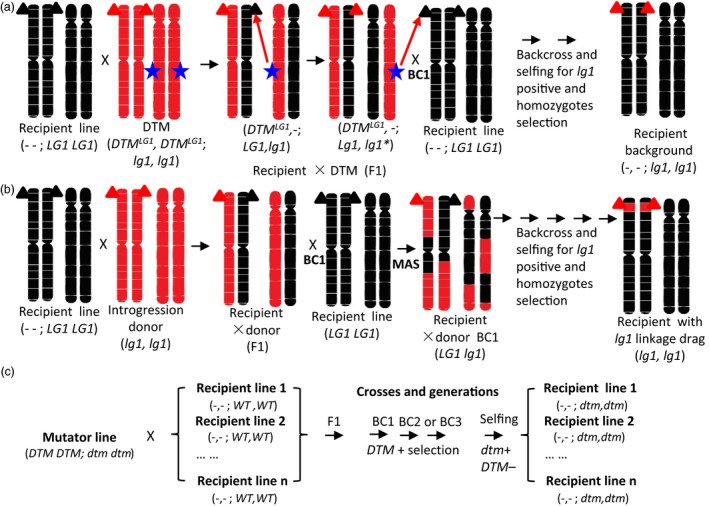
A schematic depiction of the desired‐target mutator (DTM) strategy. (a) DTM strategy. (b) The conventional backcross introgression strategy. (c) A schematic illustration of the rapid spreading of the desired‐targeted mutation (*dtm*) among recipient lines and via crossing with an RGEN mutator line during selection in breeding practice. Only two chromosome pairs with black and red colour indicating different genetic background from the plants are shown for the purpose of illustration. ‐ ‐, missing genotype; BC, backcross; black triangle, wild‐type allele; blue star, *DTM* ^*LG*^ ^*1,*^ RNA‐guided Cas9‐based RGEN targeting *ZmLG1*;* dtm*, desired‐target mutation; *DTM*, desired‐target mutator of RGEN; *DTM−*, *DTM* absence selection; *dtm*+, desired‐target mutation positive selection; *DTM* +, *DTM* presence selection; *DTM* ^*LG*^ ^*1*^, RNA‐guided Cas9‐based RGENs;MAS, marker‐assisted selection for *lg1* should be implemented during all generations; multiplication sign (×), crossing; red triangle, knockout of *lg1* allele.

**Figure 2:**
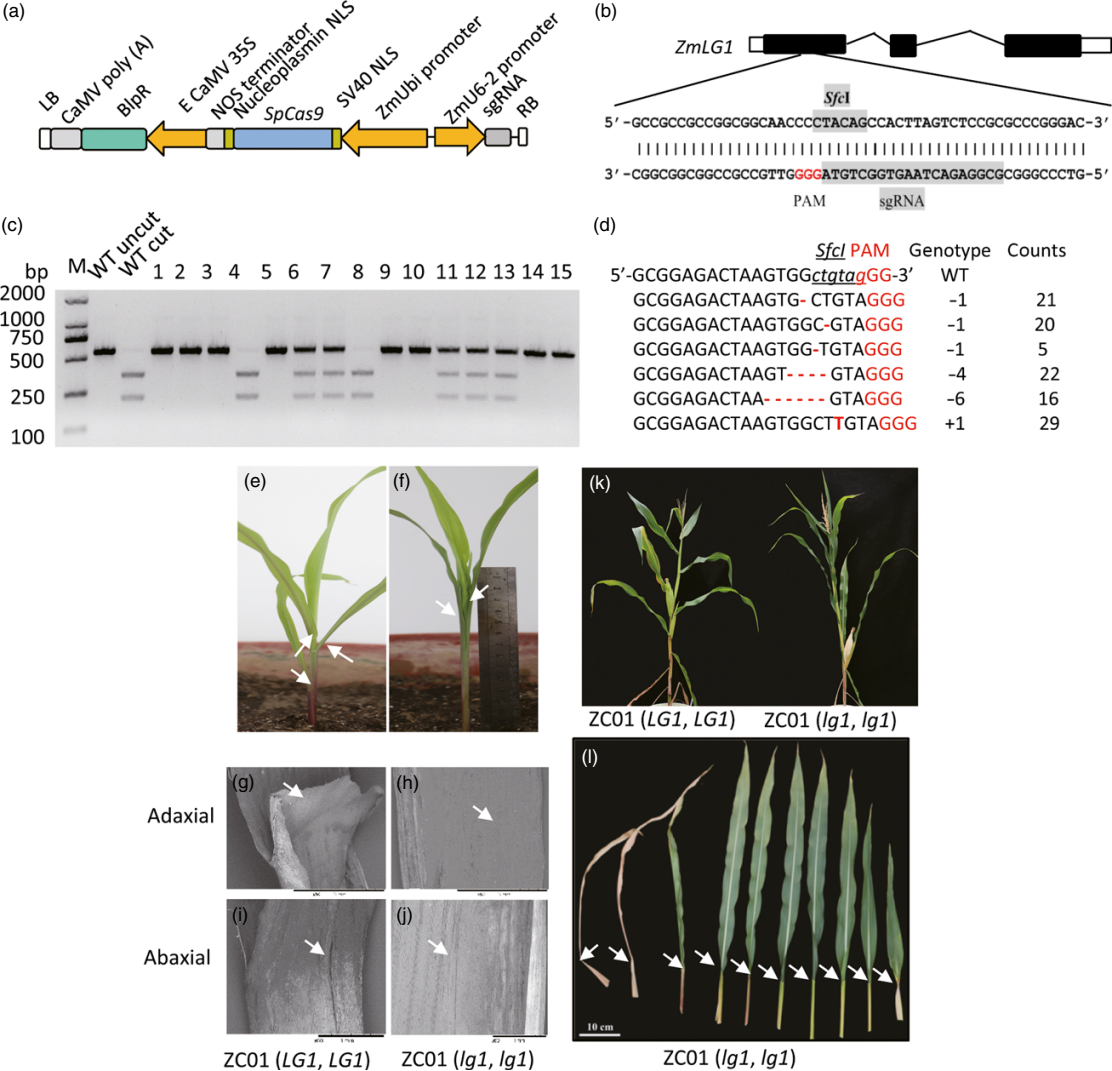
Editing of the *LG1* gene to confer upright leaves and a compact maize plant architecture. (a) Construction of the expression cassette for RNA‐guided Cas9 targeted genes. (b) The sgRNA mediating site is indicated in exon 1 within the gene structure of *ZmLG1*. The *Sfc*I restriction enzyme recognition sequence was selected within the designed mutated region of the Cas9 nuclease. The sequence of the single guiding RNA (sgRNA) region is shaded in grey. (c) PCR‐RE (*Sfc*I) assay (marker) profiles for 15 randomly selected T0 sample plants. The wild‐type sequence should be cut into two bands (WT cut). In samples 1, 2, 3, 5, 9, 10, 14 and 15, both copies of *LG1* were mutated were not cleaved. Samples 4 and 8 harboured the wild‐type allele. Samples 6, 7, 11, 12 and 13 were heterozygous for the wild‐type and targeted mutant genotypes (*LG1*,* lg1*). M, Tiangen D2000 2 K DNA marker (Tiangen, Beijing, China). (d) Relatively high frequency of targeted mutation events (*n* > 5) among 207 editing events. (e) Phenotype of a 2‐week‐old wild‐type ZC01 seedling (*LG1*, *LG1*); (f). Phenotype of the generated *LG1*‐null mutation seedling with the same genetic background as the wild‐type seedling, ZC01 (*lg1*,* lg1*). (g–j). SEM of the adaxial (g, h) and abaxial (i, j) surfaces of the junction of the sheath and leaf of the 2‐week‐old plant showing the ligule and auricle phenotypes. The wild‐type plant (g and i) exhibited a ligule and auricle, but the generated mutant (h and j) lacked both a ligule and auricle. The upright leaves and compact plant architecture were evident throughout the growing stage until the late stage (k), due to the flat angle between the sheath and leaves that resulted from ligule and auricle mutation (l).

Figure 1 is a schematic description of the RGEN design targeting maize *LG1* locus. To achieve the goal of DTM, an RNA‐guided Cas9 expression vector targeting the *LG1* gene was constructed (Figure 2a). Approximately 5000 immature ZC01 embryos from healthy maize were selected and underwent *Agrobacterium*‐mediated transformation under highly stringent bialaphos selection. In total, 113 independent transformation‐positive plants were screened and identified from around 1250 regenerated plants based on PCR with oligonucleotide primers to amplify both the *Bar* and *SpCas* genes. The transformation rate was approximately 2.26% under bialaphos selection. The relative exogenous gene copy numbers in the transformants were also evaluated; a total of 97 of 113 (85.8%) transformants harboured one to three copies of the gene (Table S1).

### Sequencing of the target regions identified mutated DNA sequences

To make the detection of mutant plants easier, we took advantage of a *Sfc*I restriction site on the single guide RNA (sgRNA) target and used it to detect mutated sites by *LG1* RGEN (Figure 2b). By digesting the PCR products containing the above region using *Sfc*I, plants of wild type, the biallelically mutated and monoallelically mutated can be distinguished by three different restriction patterns (Figure 2c). This method can also be used to detect the *LG1* mutant plant during molecular breeding.

As shown in Figure 2d and Table S1, most of the mutations were 1‐bp deletions or 1‐bp insertions. Over 90% (91.2%, 103/113) of the RGEN transformation‐positive plants harboured mutations at the *LG1* locus (Table S1; Figure S1), and 78.64% of these plants were biallelically mutated. Among them, 54.9% (62/113) displayed mutant phenotypes with both alleles being knocked out (Table S1). The mutant lacks the ligule (Figure 2g, h) and auricle (Figure 2i, j), resulting in a compact plant type (Figure 2k) with upright leaves (Figure 2e,f,l). This phenotype is evident throughout the growing stages. However, plants with in‐frame deletion or insertion of a few amino acids retained their wild‐type phenotypes. In Table S1, for instance, plants C41 and C24 exhibited wild‐type phenotypes and were identified as having 69‐bp and 36‐bp deletion respectively, at the *LG1* gene region. It suggested that ZmLG1 protein remained functional even when there were deletions of 23 and 12 amino acids, respectively, in this region. Interestingly, four plants were identified as containing mosaic genotypes (Table S1), which exhibited a partial or complete lack of ligules and upright leaves at various positions or individual upright leaves. The data on the mosaic phenotype in combination with the genotype data indicated that the mosaic plants primarily exhibited *lg1* mutant traits.

### The mutation efficiency of *LG1*‐targeting RGEN as a desired‐target mutator

To prove that stable expression of RGEN could be implemented as an *in vivo* desired‐target mutator (DTM), we crossed T1 transgenic plants carrying the RGEN gene‐editing machinery with elite maize cultivars or with its wild‐type line, ZC01 (Tables 1, S2). A total of four T1 plants derived from two T0 events were used as male parents. In total, 618 mutant plants were generated from 2890 plants from 26 F1 populations. As shown in Tables 1 and S2, the cross of T1 plants CF13‐1 and CF31‐1 to the wild‐type plant ZC01 resulted in 21.69% and 34.29% mutant plants, respectively. On average, the mutation frequencies of the four lineages were at a range of 11.97%–28.71%. We calculated the mutation frequencies of the DTM lines that stably expressing RGEN; each line generated the consistent mutant phenotypes in F1 plants, yielding mutation frequencies that ranged from 4.35% to 43.75% (Table 1). Sequencing of the expected target regions in wild‐type recipient lines showed that they has exactly same sequence at the proto‐spacer adjacent motif (PAM) and single guide RNA (sgRNA) targeting region with ZC01 (Figure S2). Thus, the mutation frequency associated with the DTM effect was as fairly high as over 20% (Table 1) but was approximately three times lower than in T0 individuals (Figure S1). This difference may be due to differences in culture times. Specifically, the T0 plants underwent at least 6 months of callus culture and another 3 months of culture after the plant was regenerated. In contrast, the DTM effect only generated the target mutation during the maize growing stage of approximately 3 months in this study, which was three times shorter than the culture period of the T0 plants.

**Table 1:** The efficiency of DTM‐generated mutation in F1 plants

T0 line	T1	Recipient × mutator line	Mutant phenotype (*n*)	Population size (*n*)	Mutant phenotype (%)	Average (%)
CF13	CF13‐1	ZC01 × CF13‐1 (CK*)	18	83	21.69	
		B73 × CF13‐1	2	46	4.35	22.92
		Mo17 × CF13‐1	9	70	12.86	
		Huangzao4 × CF13‐1	17	39	43.59	
		Dan340 × CF13‐1	10	35	28.57	
		X178 × CF13‐1	22	76	28.95	
		Ye478 × CF13‐1	24	125	19.20	
	CF13‐8	B73 × CF13‐8	14	191	7.33	11.79
		Mo17 × CF13‐8	6	109	5.50	
		Huangzao4 × CF13‐8	45	209	21.53	
		Dan340 × CF13‐8	25	155	16.13	
		X178 × CF13‐8	19	196	9.69	
		Ye478 × CF13‐8	11	104	10.58	
CF31	CF31‐8	ZC01 × CF31‐8 (CK*)	12	35	34.29	
		B73 × CF31‐8	9	62	14.52	28.71
		Mo17 × CF31‐8	46	195	23.59	
		Huangzao4 × CF31‐8	14	32	43.75	
		Dan340 × CF31‐8	21	49	42.86	
		X178 × CF31‐8	7	33	21.21	
		Ye478 × CF31‐8	15	57	26.32	
	CF31‐9	B73 × CF31‐9	80	340	23.53	28.14
		Mo17 × CF31‐9	46	176	26.14	
		Huangzao4 × CF31‐9	18	87	20.69	
		Dan340 × CF31‐9	54	163	33.13	
		X178 × CF31‐9	46	126	36.51	
		Ye478 × CF31‐9	28	97	28.87	

Positive controls (CK*) of DTM effects from were made by crossing DTM lines with its genetic background line, ZC01. The more independent transformation events of DTM effect on ZC01 could be seen in Table S2.

### Zygosity of induced mutations

**Figure 3:**
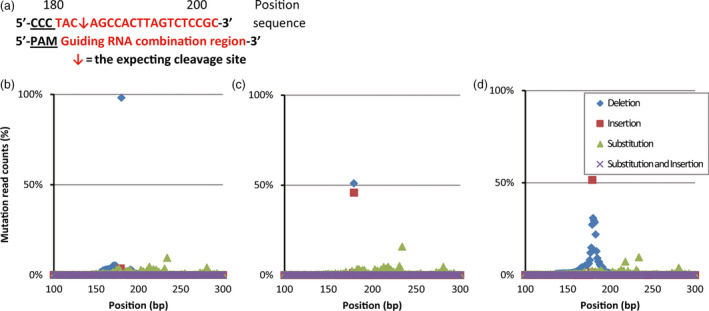
Detection of mutated loci by deep sequencing. (a) The PAM, proto‐spacer adjacent motif sequence, is underlined. The expected excision site between the 184th and 185th base pair is indicated by a red arrow between the sequence.(b) One sample plant (Dan340 × DTM‐21) harbouring a homologous 1‐bp deletion mutation (98.13% of the read counts) with very low baseline mutations surrounding the target site induced by the DTM effect. (c) One sample plant (B73 × DTM‐102) exhibiting nearly a 1 : 1 ratio of bi‐allelic mutations (50.96% 1‐bp deletions: 45.81% 1‐bp insertions) at the target site conferring the mutant phenotype induced by the targeted DTM effect in the F1 cross. (d) Sample plant (Huangzao4 × DTM‐66) exhibiting a mosaic consisting of a 1‐bp insertion (51.54%) and many mutant alleles surrounding the target site.

As *LG1* is a dominant gene, only recessive homozygotes would display mutated phenotypes, that is the upright leaves with missing ligules and auricles. Sequencing showed that among 718 *lg1* mutants as shown in Table 1, nearly all were biallelically recessive, indicating high efficiency of our DTM system. Only three plants exhibited mosaic phenotypes with partially missing ligules and auricles. To further identify the existing mutations, MiSeq‐based deep sequencing was conducted on the target regions of one mosaic phenotype Huangzao4 × DTM‐66 plant and two random selected samples showing mutant phenotype (Dan340 × DTM‐21 and B73 × DTM‐102). As saw in Figure 3, Huangzao4 × DTM‐66 was verified to harbour a chimeric mutation pattern in the target region, as demonstrated by many mutations flanking the expected DSB site. Interestingly, the pattern of the mutation rate included a sharp peak at the expected DSB site (Figure 3d), indicating that these mutations were related to RGEN mutation. Moreover, the Dan340 × DTM‐21 and B73 × DTM‐102 plants harboured a 1‐bp deletion, a bi‐allelic 1‐bp deletion and a 1‐bp insertion mutation in the target regions. The data also indicate that deep sequencing is a sensitive approach for verifying the mutations caused by RGEN.

### Inheritance and further *in vivo* editing of DTM mutations

To determine whether the targeted mutations generated by DTM were heritable, the phenotypes and mutations of selfed F2 plants were assessed. Specifically, a total of 50 F2 plants showing liguleless phenotypes were randomly selected from two F2 families (Table S3). All F2 families from F1 *lg1*‐recessive homozygote individuals showed nearly the same phenotype, except for two individuals, DW1 and HW1, which exhibited the wild‐type phenotype (Table 2). To further identify the genotypes of these individuals, the target regions of the 14 plants, including DW1 and HW1, were subjected to deep sequencing (Table 2). The results showed that DW1 and HW1 lost three or four in‐frame amino acids, respectively. Therefore, the wild‐type phenotype was restored in DW1 and HW1 because the *in vivo* DTM effect kept working in F2 plants and the originally mutated *lg1* allele was converted into a nonframe‐shift allele due to rare events. This result was consistent with our previous findings for the T0 generation (Table S1); that is, the LG1 protein can tolerate the missing or addition of small number amino acids and retain their functions.

**Table 2:** The inheritance of DTM‐generated mutations in F2 plants

ID	Selfed F1	Phenotype	Genotype	Bi‐allelic 1	Bi‐allelic 2	Mutation types	Frameshift alleles
DW1	(Dan340 × CF13‐1)F2	Wild type	*LG1 lg1*	CCCTAC—ACTTAGTCTCCGCGC	CCC——‐TTAGTCTCCGC	−4/−9	Null/3 AA missing
DW2	(Dan340 × CF13‐1)F2	Mutant	*lg1 lg1*	CCCTAC‐GCCACTTAGTCTCCGC	CCCTAC—ACTTAGTCTCCGC	−1/−4	Null/null
DW3	(Dan340 × CF13‐1)F2	Mutant	*lg1 lg1*	CCCTAC‐AGCCACTTAGTCTCCGC	CCCTACA—CTTAGTCTCCGC	−1/−4	Null/null
DW4	(Dan340 × CF13‐1)F2	Mutant	*lg1 lg1*	CCCTAC‐GCCACTTAGTCTCCGC	CCCTAC—ACTTAGTCTCCGC	−1/−4	Null/null
DW5	(Dan340 × CF13‐1)F2	Mutant	*lg1 lg1*	CCCTA—CACTTAGTCTCCGC	CCCTA—CACTTAGTCTCCGC	−4/−4	Null/null
DY1	(Dan340 × CF13‐1)F2	Mutant	*lg1 lg1*	CCCTAC‐GCCACTTAGTCTCCGC	CCCTACA‐CCACTTAGTCTCCGC	−1/−1	Null/null
DY2	(Dan340 × CF13‐1)F2	Mutant	*lg1 lg1*	CCCTAC—–TTAGTCTCCGC	CCCTAC—–TTAGTCTCCGC	−5/−5	Null/null
HW1	(Huangzao4 × CF13‐1)F2	Wild type	*lg1 lg1*	CCCTAC‐GCCACTTAGTCTCCGC	C———CACTTAGTCTCCGC	−1/−12	Null/4 AA missing
HW2	(Huangzao4 × CF13‐1)F2	Mutant	*lg1 lg1*	CCCTAC‐GCCACTTAGTCTCCGC	CCCTACAAGCCACTTAGTCTCCGC	−1/+1	Null/null
HW3	(Huangzao4 × CF13‐1)F2	Mutant	*lg1 lg1*	CCCTAC‐GCCACTTAGTCTCCGC	CCCTACAG‐CACTTAGTCTCCGC	−1/−1	Null/null
HW4	(Huangzao4 × CF13‐1)F2	Mutant	*lg1 lg1*	CCCTAC‐GCCACTTAGTCTCCGC	CCCTAC‐GCCACTTAGTCTCCGC	−1/−1	Null/null
HY1	(Huangzao4 × CF13‐1)F2	Mutant	*lg1 lg1*	CCCTAC‐GCCACTTAGTCTCCGC	CCCTAC‐GCCACTTAGTCTCCGC	−1/−1	Null/null
HY2	(Huangzao4 × CF13‐1)F2	Mutant	*lg1 lg1*	CCCTAC‐GCCACTTAGTCTCCGC	CCCTAC‐GCCACTTAGTCTCCGC	−1/−1	Null/null

## Discussion

### Improving breeding efficiency using DTM

Based on practical experience, we developed a breeding program using a RGEN as a DTM for breeding (Figure S3). Usually, at least five to six backcrosses are required to recover 99% of the elite parent genotype in maize. Although a marker‐assisted backcross (MABC) strategy is necessary under this strategy to speed up the breeding process, DTM breeding is not introgression breeding, which relies on extensive and laborious work to screen large segregating populations of thousands of individuals (Ribaut and Ragot, 2007) for background recovery in MABC programs. The DTM breeding program improved breeding efficiency in two regards. First, the target gene is directly targeted and mutated into a desirable allele via the trans‐acting function of DTM rather than by gene introgression during backcrosses. This advantage overcomes and avoids the linkage drag effect of gene introgression. Specifically, the region flanking both ends of the introgression gene is the most difficult to be recovered from the recurrent parental genome. Second, selection of the target genes is unnecessary during the breeding process. Targeted homozygous genotypes can be selected from the segregants during selfing based on their phenotype, to serve as the last generation (Figure S3). In modern maize breeding, the genetic background and foreground can be selected using molecular markers. Therefore, DTM breeding can greatly reduce the workload of background selection because it does not rely on target introgression, which is associated with linkage drag. The genetic background can be recovered from a much smaller population, and marker‐assisted selection of the target gene is unnecessary throughout the breeding process.

The targeted mutant materials developed in this study may be used in conventional backcross (introgression) breeding by crossing them with a recipient line, followed by backcrosses with the recipient line and simultaneous selection of the target genotype or trait of interest. The outcome of this ordinary backcross scheme is introgression of the target gene into the recipient line and concurrent linkage drag from the donor. Therefore, one may introduce a desired gene at the cost of introducing undesired genes. Overcoming linkage drag is laborious and requires passing through meiosis again while searching for rare recombinants between the target genes that lack the undesirable gene/QTL (Bhatia and Alok, 2014; Xu *et al*., 2014). In this breeding scheme, the mutator line is referred to as the *in vivo* mutation donor when it is crossed with the receptor (Figures 1 and S3). Targeted mutation of the receptor line can be induced in the genetic background of the receptor based on transformation events within a mutator line (such as RNA‐guided Cas9‐*LG1* in this study) rather than gene introgression, which depends on recombination during meiosis in a backcross breeding program. Thus, after selection of the expected mutation and a non‐Cas9 mutator transformation event from the backcross 1 (BC1) generation and the expected mutation in subsequent BC generations, we obtain the targeted gene mutation with 100% of the original receptor background. In contrast to conventional backcross breeding, the Cas9 mutator breeding scheme avoids the problem of linkage drag encountered during backcross breeding.

### The agronomic significance of DTM‐generated *lg1* maize lines

The high efficiency of the DTM effect of RGEN in targeting *LG1* enabled us to analyse the agronomic and breeding potential of *lg1* traits in F1 crosses for a given hybrid. Homologous *lg1* plants can be identified and separated from wild‐type plants at the early seedling stage and replanted them together in rows to evaluate their agronomic and culture potential compared with those of wild‐type rows (Table 3; Figure S4).

**Table 3:** Field characterization of agronomically related traits of DTM‐created mutant maize hybrids

Materials	Density (cm)	Leaf angle (°)	Photosynthesis response indices of the plants					Grain yield (g)	
			RCC (SPAD)	NPR (μmol m^−2^ s^−1^)	SC (mol m^−2^ s^−1^)	TR (mol m^−2^ s^−1^)	Ci (vpm)	Per plant	Per plot
Huangzao4 × DTM mutant	16 × 60	12.7 ± 9.91^a^	55.0 ± 0.57	10.14 ± 2.684^a^	0.064 ± 0.0309^a^	2.24 ± 1.229^a^	108.3 ± 6.42^b^	115.5 ± 1.23 b	11 652.3 ± 426.2^b^
Huangzao4 × DTM (CK)	16 × 60	31.6 ± 11.14	53.5 ± 1.68	6.75 ± 2.73	0.047 ± 0.0278	1.43 ± 0.996	104 ± 37.01	108.2 ± 10.52	11 425.5 ± 571.5
Huangzao4 × DTM mutant	25 × 60	12.2 ± 1.36^a^	55.5 ± 0.69	5.67 ± 2.355^a^	0.062 ± 0.007^a^	1.85 ± 0.992^a^	202.2 ± 106.87^a^	119.3 ± 5.27^a^	7637.2 ± 376.8^a^
Huangzao4 × DTM (CK)	25 × 60	29.2 ± 0.69	55.2 ± 0.98	4.83 ± 1.656	0.036 ± 0.0021	1.53 ± 0.343	174.6 ± 1.10	141.4 ± 6.46	9048.9 ± 428.3
Dan340 × DTM mutant	16 × 60	11.6 ± 2.80^a^	53.7 ± 3.36	8.36 ± 2.432^a^	0.066 ± 0.022	2.79 ± 0.76	135.4 ± 5.16^a^	94.2 ± 7.24^b^	9420.7 ± 415.6^b^
Dan340 × DTM (CK)	16 × 60	35.5 ± 0.38	51.4 ± 1.46	7.41 ± 1.43	0.069 ± 0.0291	2.85 ± 1.131	148.6 ± 18.7	92.9 ± 9.02	9288.3 ± 442.2
Dan340 × DTM mutant	25 × 60	12.2 ± 0.13^a^	51.1 ± 1.18	7.47 ± 1.129^b^	0.081 ± 0.0045	2.22 ± 0.358^a^	186.2 ± 17.18	109.6 ± 4.29^b^	7014.4 ± 384.2^a^
Dan340 × DTM (CK)	25 × 60	25.8 ± 6.38	51.9 ± 0.01	7.89 ± 2.637	0.075 ± 0.0333	1.76 ± 0.939	190.9 ± 18.46	116.8 ± 9.06	7473.3 ± 348.6

RCC, Relative chlorophyll content; NPR, net photosynthetic rate; SC, stomatal conductance; TR, transpiration rate; intercellular CO_2_ concentration (Ci).

Pairwise Student's *t*‐test of significance between the mutant and CK.

a *P *< 0.01.

b *P *< 0.05.

Important phenotypic differences in physiological and grain yield indices were identified between the mutant and wild‐type plants, with the leaf angle (LA) being reduced more than 50% (Table 3). The relative chlorophyll content of the ear leaf did not differ between mutant and wild‐type plants, but photosynthesis indices, including the net photosynthetic rate, stomatal conductance, respiratory rate and intercellular CO_2_ concentration, differed significantly between the mutant and its full‐sibling wild‐type plants. Thus, the change in plant morphology affected the photosynthetic activity of plants. Regarding grain yield, both the grain yield per plant and grain yield per plot were significantly lower (*P *< 0.01) in the mutant than in wild‐type plants at the lower planting density (25 cm row spacing). However, at a higher density (16 cm row spacing), both the grain yield per plant and grain yield per plot were significantly higher (*P *< 0.05) for mutant than for wild‐type plants. These results indicated that DTM‐created target mutation plants provided higher potential for an increased grain yield at a higher density. In fact, the potential may significantly exceed one's expectation with more than 90 000/ha plants (16 cm × 60 cm spacing), as the intensity of sunlight observed on the ground in the field at 13:00 hrs indicated that the mutant could have higher density potential to get the better field sunlight interception (Figure S5).

### Applicability of DTM, genotype‐independent editing and delivery of the editing machinery

The DTM effect enabled by genome editing shows advantages over traditional plant breeding. However, these advantages would disappear if a genome‐editing protocol became applicable in any genotype background because sexual crossing would no longer be necessary to spread the mutation. Moreover, the DTM effect may only be applicable for gene knockout and deletion mutations and may not be applicable for gene replacement, which requires a DNA repair donor (Li *et al*., 2016; Svitashev *et al*., 2015; Zhao *et al*., 2016).

Delivery of the machinery during genome‐editing protocols involves the generation of a targeted mutation via transient expression (Li *et al*., 2016; Svitashev *et al*., 2015; Zhang *et al*., 2016) or ‘DNA‐free’ ribonucleoprotein (RNP) delivery (Woo *et al*., 2015) in any genotype background, which avoids the regulatory concerns associated with genetically modified organisms (GMOs) (Huang *et al*., 2016; Woo *et al*., 2015). This approach allows us to generate a desired mutant allele in any genetic background. The delivery of genome‐editing machinery into the target cells is a key step and strategy for the generation of heritable mutations in plants. In plants, the gene‐editing machinery can be transiently expressed using biolistic transformation to penetrate the cell wall (Li *et al*., 2016). However, particle bombardment strategies may also result in extensive DNA arrangement or high copy numbers of exogenous segmentation (Register *et al*., 1994; Shou *et al*., 2004), which are a cause for concern and are frequently found in plants transformed via direct gene transfer methods (Ishida *et al*., 1996; Shou *et al*., 2004). The RNP strategy is exciting because GM problems are not a concern (Svitashev *et al*., 2016; Woo *et al*., 2015). Both transient expression and RNP methods should regenerate plants. Plant regeneration from the protoplast, suspension cells, or callus is either impractical or highly specific to the genotype and involves laborious and time‐consuming tissue culture steps in almost all major crop species, including maize, wheat, rice and soya bean (Birch, 1997; Rhodes *et al*., 1988). Most important species and major crop species have been stably transformed using a few easily transformed genotypes (Birch, 1997; Gupta and Ram, 2004), and the use of genome‐editing machinery in trans‐mode to generate an intentional mutation will remain a practical method until the technology for transient RNP gene editing is mature.

## Materials and methods

### Construction of the RNA‐guided Cas9 vector

The modified coding sequence of *SpCas9* (Cong *et al*., 2013) was cloned into the CPB vector behind a maize ubiquitin promoter. The *SpCas9* region was amplified and cloned into the CPB vector using the pEASY^®^‐Uni Seamless Cloning and Assembly Kit (Transgene, CU101, Beijing, China). The nuclear location signal (NLS) sequence of SV40 and nucleoplasmin were embedded at either end of the Cas9 protein. The guiding RNA sequence 5′‐GCGGAGACTAAGTGGctgtagGG‐3′, which harbours an *Sfc*I region (lowercase region), was selected to target the maize *LG1* locus within the coding region of exon 1 at chromosome 2 from 4265163 to 4268840 (AGP v3.0). The underlined ‘gGG’ represents the proto‐adjacent‐motif (Figure 2). The *maize* U6‐6 promoter was used to drive the sgRNA gene, and the promoters and sgRNA genes were cloned into the CPB vector following the manufacturer's suggested protocols. The sequences of the key elements employed in this study are listed in detail in the Table S4.

### Maize transformation

The high‐efficiency transformation of ZC01 maize, a private receptor inbred line, was conducted by the China National Seed Group Co., LTD (Wuhan, China), based on a modified *Agrobacterium tumefaciens* (EHA105 strain)‐mediated immature embryo transformation protocol (Ishida *et al*., 1996). Briefly, the *Agrobacterium* EHA105 strain was used for transformation, and transformed cells were selected for 2 weeks using 5 mg/L bialaphos (Sigma‐Aldrich, St. Louis), followed by selection with 8 mg/L bialaphos for 2 weeks.

Genomic DNA was analysed using quantitative real‐time PCR (qPCR) targeting the Bar gene and the SYBR Green method (Roche Cat. No. 04913914001) to determine transgene copy numbers. The primer pair targeting the Bar gene was 5′‐CAGGAACCGCAGGAGTGGAC‐3′ (forward primer) and 5′‐CTTCAGCAGGTGGGTGTAGAGC‐3′ (reverse primer). The endogenous *ivr* gene was employed as the single‐copy reference gene and was analysed using the following primers: 5′‐ACTAGGCATCCAAGGCGAACG‐3′ (forward primer) and 5′‐AGTGCGAGAAGAA CGAGTGTCC‐3′ (reverse primer). The PCR cycling parameters were as follows: 95 °C for 10 min and 35 cycles of 95 °C for 10 s, 60 °C for 55 s and 72 °C for 30 s. *Bar* gene expression was quantified and normalized to *ivr* gene expression using the 2^−ΔΔCt^ method. Only reactions showing a PCR efficiency greater than 90% were analysed.

### DNA extraction and PCR‐RE assay

DNA was isolated and purified using an absorption column method and the DNeasy plant mini kit (Qiagen, Germany) according to the manufacturer's protocols. Genomic DNA was extracted from maize T0, T1 and F1 (mutator crossed with recipient lines) *lg1* mutants, and the on‐target sites were amplified from genomic DNA. The amplicons were designed to surround the intended target site, and PCR was conducted using high‐fidelity KOD‐plus polymerase (Catalog#: KOD‐401, TOYOBO Life Science Depart., Osaka, Japan) and primers spanning the target sites. The primer pairs used for PCR were 5′‐GCGTGGGAAGATGATGAACC‐3′ and 5′‐GTACGTGTAGCCTCCTCTGG‐3′. PCR was carried out in a 50 μl reaction on a Bio‐Rad T100 instrument (Bio‐Rad, Hercules, CA), as follows: denaturation at 94 °C for 2 min; 35 cycles of 94 °C for 15 s, 62.3 °C for 30 s and 68 °C for 40 s; and a final extension at 68 °C for 10 min. The amplicons were then purified using a kit (catalog#: AP‐GX‐250, Axygen, NY) and digested with *Sfc*I following the kit's instructions. The restriction site was located at the cleavage site of RNA‐guided Cas9, and a mutation changes the RE restriction pattern, which allowed mutants to be quickly distinguished from wild‐type individuals. The RE restriction products were visualized in agarose gels.

### Sanger sequencing to identify the target mutation

Sanger sequencing was used to identify the mutation in the T0 generation. The PCR amplicons obtained from the samples were cloned into the pCR TA clone vector using a commercial kit (Transgene, Beijing China) based on the provided protocols and procedures. The M13 R primer was used for sequencing in an ABI3730 instrument (Applied Biosystems, California) to identify the mutations. The sequencing quality and results were viewed using Sequence Scanner Software ver2.0 (ABI Applied Biosystems) by importing the raw sequencing trace files. The homologous mutant genotypes of T0 individuals were cross‐validated across the DNA samples extracted from seedling leaves, young male inflorescences, emerging silk and young ear husks. Mutations were identified in each sample of T0 transformation events based on at least 20 independent high‐quality positive sequences from TA clones carrying the PCR amplicons.

### The target region sequence of the recipient lines

The ZC01 and six recipient lines was amplified and sequenced. These data are given in Table S4. The sequence six recipient lines, B73, Mo17, Huangzao4, Dan340, X178 and Ye478, had been deposited in GenBank under the accessions KY607009, KY607010, KY607011, KY607012, KY607013 and KY607014 with Bankit ID 1989559. The alignment of sequences was showed on Figure S2.

### Mutant phenotype scoring and scanning electron microscopy

The ligules and auricles of maize leaves were scored via visual examination because they present visible phenotypes. Scanning electron micrographs of the ligules and auricles of mutant and wild‐type maize leaves were obtained on a NOVA NanoSEM 430 (FEI) scanning electron microscope according to the provided protocols. Briefly, freshly dissected maize leaves were fixed overnight and then dehydrated with ethanol and critical‐point dried with liquid CO_2_. The samples were subsequently coated with powered gold for examination.

### DTM crossing and DTM mutation rate

To test the DTM effect, four T1 lines harbouring one homologous copy of the *LG1*‐targeted knockout editing machinery transformant were selected (Table 1) as pollen donors to be crossed with the inbred lines B73, Mo17, Huangzao4, Dan340, X178 and Ye478. Also, the different DTM events were planted for selfing or crossing with its’ own wild type for seed increase. For hand pollination, at least five ears, those were on the fifth day after silks first emerged from the husks and prevented from the outcross before silking by bag, of each recipient were artificial pollinated with fresh pollen from donor between 11:00 am and 14:00 pm. These recipient lines exhibit diverse genetic bases (Xie *et al*., 2007). The F1 hybrids, crosses between the recipient lines and DTM, were planted in a goblet block until the fourth true leaves emerged, which were used to assess the ligule and auricle phenotypes and to calculate the DTM mutation rate, as the mutant phenotype was only evident when the *LG1* allele from the recipient line had mutated into the targeted recessive allele.

### Deep sequencing of the target PCR amplicon and zygosity of DTM‐induced mutations

Deep sequencing was employed to identify the mutations induced by the DTM effect. To verify that the mutations were heritable, partial inflorescences from young males and newly emerging young ear silks were sampled. Both male and female inflorescences were covered with a glassine bag before emergence to prevent contamination by insects and pollen from neighbouring plants. The plant tissues were mixed for DNA isolation, PCR amplification and deep sequencing. The resulting PCR amplicons were purified with a Qiagen PCR purification spin column (Qiagen, Germany), and the DNA concentration was measured in a Hoechst dye‐based fluorometric assay. The samples were combined at an equimolar ratio, and single‐read 100‐nucleotide‐long amplicon sequencing was performed in an Illumina MiSeq Personal Sequencer with a 30%–40% (v/v) spike of PhiX control v3 (Illumina, FC‐110‐3001) to offset sequence bias. Only reads with ≥1 nucleotide indel arising within the 10 nt window centred over the expected site of cleavage that were not found in the negative controls were classified as NHEJ mutations. NHEJ mutant reads with the same mutation were counted and collapsed into a single read, and the 10 most prevalent mutations were visually confirmed as arising within the expected site of cleavage. The total number of visually confirmed mutations was then used to calculate the percentage of mutant reads. The threshold percentage for mutations in clean reads was set to lower than 3%, which is regarded as the background mutation rate or the rate of rare mutations determined by tracing reversion mutations induced by DTM.

### Field planting and management

Six mutant F1 hybrids were replanted in a field in spring, 2016, at the Shunyi Experimental Station of the Institute of Crop Science of the Chinese Academy of Agricultural Sciences (40.2°N 116.5°E, 44 MASL elevation). Two densities, with plant spacing of 16 cm (high density) or 25 cm (low density) between within each row and spacing of 60 cm between rows, were applied following a split‐plot design with two replicates. Each plot included four rows, and each row was 4 m in length. Twenty‐five and 16 individuals were planted in the high‐density and low‐density rows, respectively. The field was managed according to a routine experimental procedure for growing maize. Briefly, 80.5 kg of nitrogen (N) ha^−1^ as urea, 51 kg of phosphorous ha^−1^ as calcium superphosphate, and 90 kg of potassium ha^−1^ as muriate of potash were applied as fertilizer before planting, and a second application of 161 kg N ha^−1^ and 90 kg of potassium ha^−1^ top fertilizer was performed at the maize V8 stage. Water irrigation and pest control were conducted in accordance with the routine protocols of the experimental station.

### Field experiment and trait measurements

At the R2 growth stage, the chlorophyll content was measured using a SPAD502 instrument (Minolta Camera Co. Ltd., Japan). The net photosynthetic rate, stomatal conductance, intercellular CO_2_ concentration and transpiration rate were measured using the Lcpro+ Ultra Compact Photosynthesis System (ADC BioScientific Ltd., Herts, England). Three leaves (the third leaf above the uppermost ear, the ear leaf and the third leaf below the uppermost ear) were measured three times, and three randomly selected plants were measured in each row. The leaf angle (LA) was determined as previously described (Ku *et al*., 2010). Seven leaves (the three leaves above the uppermost ear, the leaf at the ear position and the three leaves below the dominant ear) were employed to measure the LA.

To avoid the marginal effect, the ears of the plants in the two middle rows except for the two plants at both row ends of each plot were harvested at the R6 growth stage and used to quantify the grain yield per plant and grain yield per plot. The grain yield per plot was normalized using the average plant grain yield for four plants in a row.

### Data analysis

One‐way ANOVA with split‐plot design was performed to analyse and compare each trait between mutant hybrid and wild hybrid using GLM (general linear model) on R version 3.3.0 (R Core Team, 2016). Pairwise Student's *t*‐test comparison (*P < 0.05; and **P < 0.01) between the mean value of each pair of mutant hybrid and wild hybrid had been performed on Excel2013.

## Conflict of interest

The authors declare no conflict of interest.

## Supporting information


**Figure S1** Summary of mutation types and frequencies among 113 T0 transformation events.
**Figure S2** The alignment of the DTM targeting DNA region along with the flanking sequence of the 6 recipient lines (B73, Mo17, Huangzao4, Dan340, X178, and Ye478).
**Figure S3** A DTM breeding program designed based on experience accelerated the spreading of the mutation and genetic background recovery.
**Figure S4** The desired‐target mutation of the *lg1* homologous mutant phenotype induced by crossing with ZC01 DTM (F1), which allowed an increased planting density and resulted in increased production potential among the different recipient lines.
**Figure S5** Density potential of the DTM‐generated F1 hybrid.

**Table S1** Identification of the target mutation among 113 T0 transformants along with the mutation frequencies and phenotype.
**Table S2** Frequency of mutations induced by the *in vivo* DTM effect resulting in an intended mutant phenotype among the progeny of crosses with its’ wild type genetic background.
**Table S3** Validation of the heritability of mutation induced by DTM effect in the successive F2 generation of 2 F1 crosses.
**Table S4** The sequence of the mentioned key elements of RNA‐guided Cas9 expression cassette in this study.
