# Supplementary material for: RNA‐guided Cas9 as an in vivo desired‐target mutator in maize
Source: Plant Biotechnol J. 2017 May 12;15(12):1566–76. doi: 10.1111/pbi.12739 (PMC5698053; doi:10.1111/pbi.12739)
Supplement: Supplementary file 1 — Figure S1 Summary of mutation types and frequencies among 113 T0 transformation events. Figure S2 The alignment of the DTM targeting DNA region along with the flanking sequence of the 6 recipient lines (B73, Mo17, Huangzao4, Dan340, X178, and Ye478). Figure S3 A DTM breeding program designed based on experience accelerated the spreading of the mutation and genetic background recovery. Figure S4 The desired‐target mutation of the lg1 homologous mutant phenotype induced by crossing with ZC01 DTM (F1), which allowed an increased planting density and resulted in increased production potential among the different recipient lines. Figure S5 Density potential of the DTM‐generated F1 hybrid. [file PBI-15-1566-s001.docx]

Supplementary figures (Figure S1-S5)

**Figure S1. Summary of mutation types and frequencies among 113 T0 transformation events.**

Note, ** The frequency of individuals with the mutant phenotype was much higher than the frequency of homologous mutant genotypes identified by sequencing because individuals may contain different mutant alleles from bi-allelic and mosaic plants.

**Figure S 2. The alignment of the DTM targeting DNA region along with the flanking sequence of the 6 recipient lines (B73, Mo17, Huangzao4, Dan340, X178, and Ye478).**

The sgRNA/Cas9 targeting region was indicated with a red arrow.

The partial *ZmLG1* locus sequence (longer than this alignment) of 6 recipient lines had been deposited in GenBank under the accessions and Bankit ID below

- BankIt1989559 B73-LG1-partial KY607009
- BankIt1989559 Mo17-LG1-partial KY607010
- BankIt1989559 Huangzao4-LG1-partial KY607011
- BankIt1989559 Dan340-LG1-partial KY607012
- BankIt1989559 X178-LG1-partial KY607013
- BankIt1989559 Ye478-LG1-partial KY607014

**
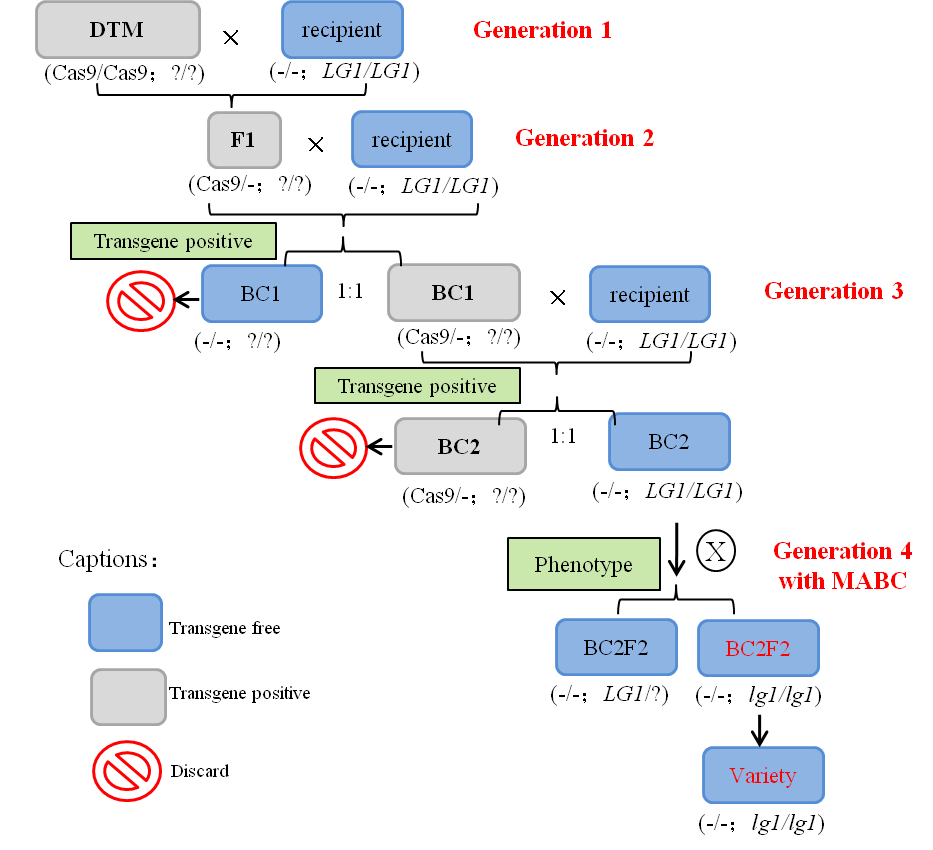
**

**Figure S3. A DTM breeding program designed based on experience accelerated the spreading of the mutation and genetic background recovery.**

?/?: irrespective of the target, wild-type or mutant allele; /?, irrespective of the wild-type or mutant allele; -/-; missing genotypes; DTM, desired-target-knockout mutation; Cas9/Cas9；effective CRISPR/Cas9 transformation machinery

Plants positive for the transgene could be identified using a lab-independent toolkit (i.e., LibertyLink® (*bar*) (Envirologix, USA).


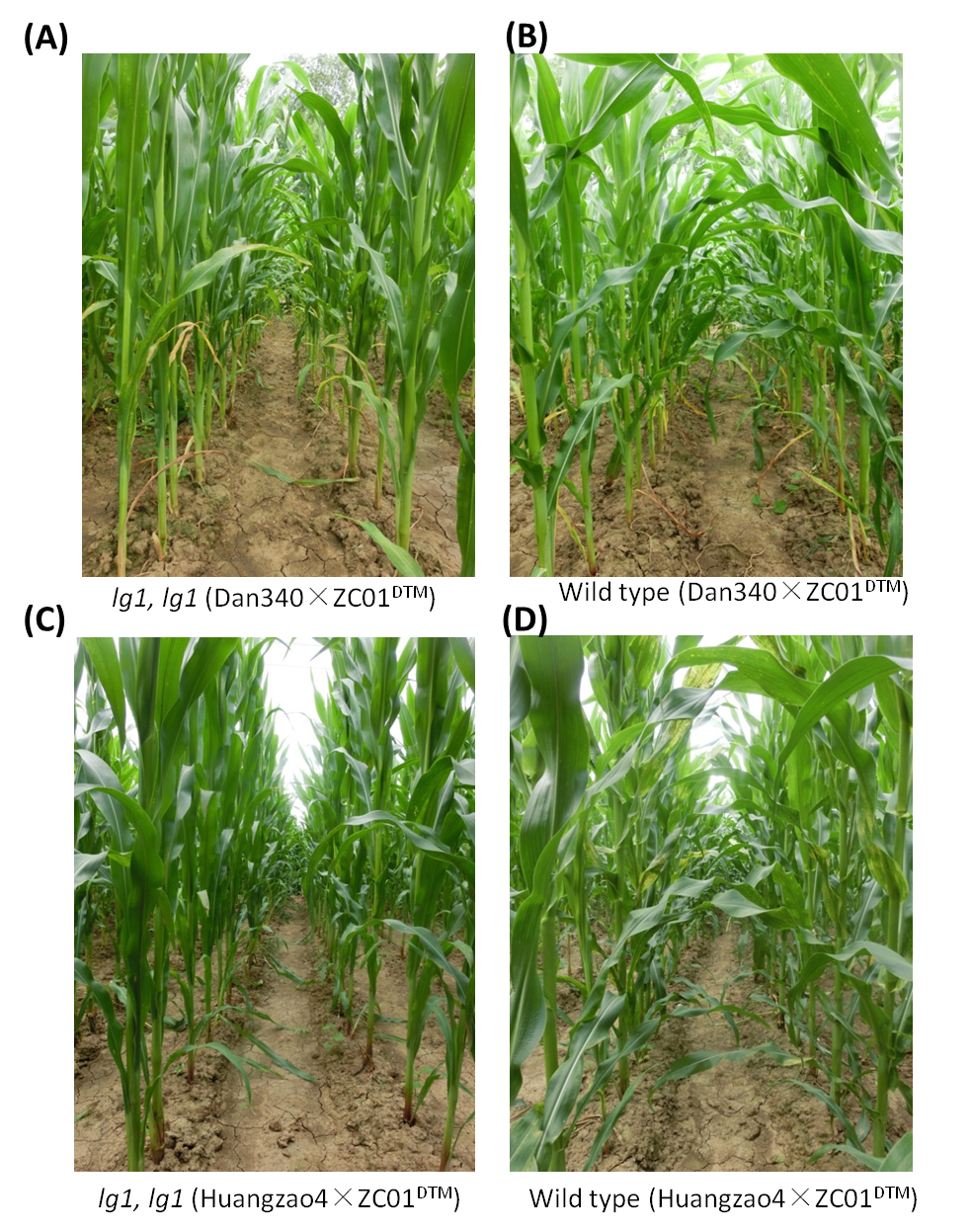


**Figure S4. The desired-target mutation of the consistent *lg1* mutant phenotype induced by crossing with ZC01^DTM^ (F1), which allowed an increased planting density and resulted in increased production potential among the different recipient lines.**

(A) *lg1, lg1* (Dan340×ZC01^DTM^); (B) ck, *Wild type* (Dan340×ZC01^DTM^); (C) *lg1, lg1* (Huangzao4×ZC01^DTM^); (D) Wild type (Huangzao4×ZC01^DTM^). The recipient maize inbred lines Dan340 and Huangzao4 were crossed with the ZC01^DTM^ line, which can induce the desired knockout mutation at *LG1*, the targeted locus. The F1 seedlings were germinated, and the homologous mutants and wild-type plants were then selected and planted in separate rows in fields. Mutant plants could be planted at a higher density because they exhibited upright leaves and compact architecture.


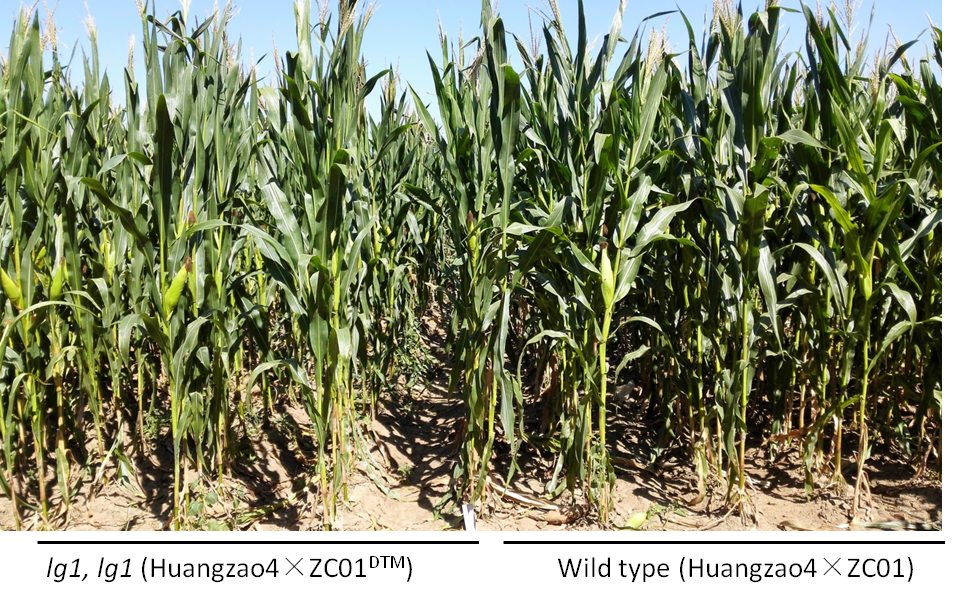


**Figure S 5. Density potential of the DTM-generated F1 hybrid.**

The mutant hybrid induced by the DTM effect (Huangzao4×ZC01^DTM^) could be planted at higher densities than wild-type plants, of up to 90,000 plants/ha ( 16 cm × 60 cm spacing), because the mutant had more access to light, as indicated by the intensity of sunlight reaching the ground in the field around 13:00 PM.
