# Supplementary material for: RNA‐guided Cas9 as an in vivo desired‐target mutator in maize
Source: Plant Biotechnol J. 2017 May 12;15(12):1566–76. doi: 10.1111/pbi.12739 (PMC5698053; doi:10.1111/pbi.12739)
Supplement: Supplementary file 2 — Table S1 Identification of the target mutation among 113 T0 transformants along with the mutation frequencies and phenotype. Table S2 Frequency of mutations induced by the in vivo DTM effect resulting in an intended mutant phenotype among the progeny of crosses with its’ wild type genetic background. Table S3 Validation of the heritability of mutation induced by DTM effect in the successive F2 generation of 2 F1 crosses. Table S4 The sequence of the mentioned key elements of RNA‐guided Cas9 expression cassette in this study. [file PBI-15-1566-s002.docx]

**Table S1. Identification of the target mutation among 113 T0 transformants along with the mutation frequencies and phenotype.**

| **ID** | **Pheno.** | **Elisa/Cas9** | **Relative copies** | **Zygosity** | **Allele 1 mutation** | **Allele 2 mutation** | **Mutation Zygosity** |
| --- | --- | --- | --- | --- | --- | --- | --- |
| A01 | mutant | +/+ | 4.0 | heterozygote | +1 | -7 | bi-allelic |
| A02 | WT | +/+ | 1.0 | heterozygote | -6 | -2 | bi-allelic |
| A03 | WT | +/+ | 1.0 | homozygote | wild type | wild type | not detected |
| A04 | WT | +/+ | 19.0 | heterozygote | -6 | -4 | bi-allelic |
| A05 | WT | +/+ | 1.0 | heterozygote | -3 | +1 | bi-allelic |
| A06 | mutant | +/+ | 3.0 | heterozygote | -2 | -1 | bi-allelic |
| A07 | WT | +/+ | 2.0 | heterozygote | -3 | -4 | bi-allelic |
| A08 | mutant | +/+ | 4.0 | heterozygote | -11 | +1 | bi-allelic |
| A09 | WT | +/+ | 1.0 | heterozygote | -6 | -1 | bi-allelic |
| A10 | WT | +/+ | 1.0 | homozygote | wild type | wild type | not detected |
| A11 | mutant | +/+ | 1.0 | homozygote | -1 | homozygote | homozygote |
| A12 | WT | +/+ | 1.0 | homozygote | wild type | wild type | not detected |
| A13 | mutant | +/+ | 4.0 | heterozygote | +1 | -1 | bi-allelic |
| A14 | WT | +/+ | 1.0 | heterozygote | -602 | -57 | bi-allelic |
| A15 | mutant | +/+ | 10.0 | heterozygote | -2 | -2 | bi-allelic |
| A16 | WT | +/+ | 2.0 | homozygote | wild type | wild type | not detected |
| A17 | WT | +/+ | 6.0 | heterozygote | -3 | -1 | bi-allelic |
| A18 | mutant | +/+ | 3.0 | heterozygote | -1 | +180 | bi-allelic |
| A19 | WT | +/+ | 2.0 | heterozygote | -9 | -3 | bi-allelic |
| A20 | WT | +/+ | 3.0 | heterozygote | -4 | -3 | bi-allelic |
| A21 | mutant | +/+ | 1.0 | heterozygote | -4 | +1 | bi-allelic |
| A22 | WT | +/+ | 3.0 | heterozygote | -7 | -6 | bi-allelic |
| A23 | WT | +/+ | 1.0 | homozygote | -6 | homozygote | homozygote |
| A24 | mosaic | +/+ | 1.0 | mosaic | multiple | multiple | mosaic |
| A25 | WT | +/+ | 2.0 | homozygote | wild type | wild type | not detected |
| A26 | WT | +/+ | 4.0 | heterozygote | -1 | -9 | bi-allelic |
| A27 | WT | +/+ | 1.0 | heterozygote | -6 | -1 | bi-allelic |
| A28 | WT | +/+ | 6.0 | homozygote | -3 | homozygote | homozygote |
| A29 | WT | +/+ | 1.0 | homozygote | wild type | wild type | not detected |
| A30 | WT | +/+ | 1.0 | heterozygote | -25 | -12 | bi-allelic |
| A31 | WT | +/+ | 3.0 | heterozygote | -1 | -6 | bi-allelic |
| A32 | mutant | +/+ | 3.0 | homozygote | +1 | homozygote | homozygote |
| A33 | WT | +/+ | 2.0 | heterozygote | -6 | -4 | bi-allelic |
| B01 | mutant | +/+ | 1.0 | heterozygote | -7 | -4 | bi-allelic |
| B02 | mutant | +/+ | 3.0 | heterozygote | -11 | -8 | bi-allelic |
| B03 | mutant | +/+ | 3.0 | homozygote | -43 | homozygote | homozygote |
| B04 | mutant | +/+ | 1.0 | homozygote | -1 | homozygote | homozygote |
| B05 | mutant | +/+ | 7.0 | heterozygote | -6 | -4 | bi-allelic |
| B06 | WT | +/+ | 16.0 | heterozygote | -12 | -4 | bi-allelic |
| B07 | mutant | +/+ | 2.0 | heterozygote | -11 | -17 | bi-allelic |
| **ID** | **Pheno.** | **Elisa/Cas9** | **Relative copies** | **Zygosity** | **Allele 1 mutation** | **Allele 2 mutation** | **Mutation Zygosity** |
| B08 | mutant | +/+ | 1.0 | heterozygote | -5 | -2 | bi-allelic |
| B09 | mutant | +/+ | 3.0 | heterozygote | -5 | -5 | bi-allelic |
| B10 | mutant | +/+ | 5.0 | heterozygote | -8 | -4 | bi-allelic |
| B11 | mutant | +/+ | 1.0 | heterozygote | +115 bp duplication +6 insertion -6 deletion | -1 | bi-allelic |
| B12 | WT | +/+ | 6.0 | heterozygote | -6 | +1 | bi-allelic |
| B13 | mutant | +/+ | 22.0 | heterozygote | -1 | -4 | bi-allelic |
| B14 | mutant | +/+ | 4.0 | heterozygote | -1 | +1 | bi-allelic |
| B15 | mutant | +/+ | 3.0 | homozygote | +1 | homozygote | homozygote |
| C01 | mutant | +/+ | 1.0 | heterozygote | +1 | -4 | bi-allelic |
| C02 | mutant | +/+ | 1.0 | homozygote | -1 | homozygote | homozygote |
| C03 | mutant | +/+ | 1.0 | heterozygote | -4 | +2 | bi-allelic |
| C04 | WT | +/+ | 1.0 | heterozygote | 1 substitution | -4 | bi-allelic |
| C05 | WT | +/+ | 1.0 | heterozygote | -6 | -11 | bi-allelic |
| C06 | mutant | +/+ | 1.0 | homozygote | +1 | homozygote | homozygote |
| C07 | WT | +/+ | 1.0 | homozygote | -6 | homozygote | homozygote |
| C08 | mutant | +/+ | 2.0 | heterozygote | -155 | -16 | bi-allelic |
| C09 | WT | +/+ | 1.0 | heterozygote | wild type | -6 | bi-allelic |
| C10 | mutant | +/+ | 4.0 | heterozygote | -17 | -11 | bi-allelic |
| C11 | WT | +/+ | 1.0 | homozygote | -6 | homozygote | homozygote |
| C12 | mutant | +/+ | 1.0 | heterozygote | -1 | -8 | bi-allelic |
| C13 | WT | +/+ | 1.0 | homozygote | wild type | wild type | not detected |
| C14 | WT | +/+ | 1.0 | heterozygote | -3 | -6 | bi-allelic |
| C15 | mutant | +/+ | 1.0 | heterozygote | -1 | -4 | bi-allelic |
| C16 | mutant | +/+ | 1.0 | heterozygote | -4 | -1 | bi-allelic |
| C17 | WT | +/+ | 2.0 | heterozygote | -4 | 1 subsititution | bi-allelic |
| C18 | WT | +/+ | 1.0 | heterozygote | -33 | -3 | bi-allelic |
| C19 | mutant | +/+ | 4.0 | heterozygote | +11 | -4 | bi-allelic |
| C20 | mutant | +/+ | 1.0 | heterozygote | -5 | -4 | bi-allelic |
| C21 | mutant | +/+ | 2.0 | heterozygote | +1 | -5 | bi-allelic |
| C22 | mutant | +/+ | 2.0 | heterozygote | -2 | -1 | bi-allelic |
| C23 | mutant | +/+ | 1.0 | homozygote | -91 | homozygote | homozygote |
| C24 | WT | +/+ | 2.0 | heterozygote | -43 | -36 | bi-allelic |
| C25 | WT | +/+ | 1.0 | heterozygote | -5 | -6 | bi-allelic |
| C26 | mosaic | +/+ | 1.0 | mosaic | multiple | multiple | mosaic |
| C27 | WT | +/+ | 2.0 | heterozygote | -6 | -1 | bi-allelic |
| C28 | mosaic | +/+ | 1.0 | mosaic | multiple | multiple | mosaic |
| C29 | WT | +/+ | 1.0 | heterozygote | -12 | -2 | bi-allelic |
| **ID** | **Pheno.** | **Elisa/Cas9** | **Relative copies** | **Zygosity** | **Allele 1 mutation** | **Allele 2 mutation** | **Mutation Zygosity** |
| C30 | mutant | +/+ | 1.0 | homozygote | -1 | homozygote | homozygote |
| C31 | mutant | +/+ | 1.0 | heterozygote | -1 | -4 | bi-allelic |
| C32 | mutant | +/+ | 1.0 | heterozygote | +1 | -4 | bi-allelic |
| C33 | mutant | +/+ | 1.0 | heterozygote | -1 | -1 | bi-allelic |
| C34 | WT | +/+ | 1.0 | heterozygote | -3 | -1 | bi-allelic |
| C35 | mutant | +/+ | 1.0 | heterozygote | -7 | -1 | bi-allelic |
| C36 | WT | +/+ | 1.0 | heterozygote | -3 | -6 | bi-allelic |
| C37 | mutant | +/+ | 1.0 | heterozygote | +1 | -2 | bi-allelic |
| C38 | mutant | +/+ | 1.0 | heterozygote | -10 | -1 | bi-allelic |
| C39 | WT | +/+ | 1.0 | heterozygote | -11 | -3 | bi-allelic |
| C40 | WT | +/+ | 1.0 | homozygote | wild type | wild type | not detected |
| C41 | WT | +/+ | 1.0 | heterozygote | -69 | -1 | bi-allelic |
| C42 | mutant | +/+ | 1.0 | heterozygote | -2 | +1 | bi-allelic |
| C43 | mutant | +/+ | 1.0 | heterozygote | -8 | -1 | bi-allelic |
| C44 | mutant | +/+ | 1.0 | homozygote | +1 | homozygote | homozygote |
| C45 | mutant | +/+ | 1.0 | homozygote | +1 | homozygote | homozygote |
| C46 | mutant | +/+ | 1.0 | heterozygote | -1 | +1 | bi-allelic |
| C47 | mutant | +/+ | 1.0 | homozygote | -1 | homozygote | homozygote |
| C48 | mutant | +/+ | 1.0 | heterozygote | +1 | -1 | bi-allelic |
| C49 | WT | +/+ | 1.0 | homozygote | wild type | wild type | not detected |
| C50 | mutant | +/+ | 1.0 | homozygote | -1 | homozygote | homozygote |
| C51 | mutant | +/+ | 2.0 | homozygote | +1 | homozygote | homozygote |
| C52 | WT | +/+ | 1.0 | heterozygote | -25 | -6 | bi-allelic |
| C53 | WT | +/+ | 1.0 | heterozygote | -1 | -6 | bi-allelic |
| C54 | mutant | +/+ | 1.0 | homozygote | +1 | homozygote | homozygote |
| C55 | mutant | +/+ | 1.0 | heterozygote | -1 | -4 | bi-allelic |
| C56 | mutant | +/+ | 1.0 | heterozygote | +1 | -1 | bi-allelic |
| C57 | mutant | +/+ | 1.0 | heterozygote | +1 | -2 | bi-allelic |
| C58 | mosaic | +/+ | 1.0 | mosaic | multiple | multiple | mosaic |
| C59 | WT | +/+ | 1.0 | heterozygote | -3 | -17 | bi-allelic |
| C60 | WT | +/+ | 1.0 | heterozygote | +3 | -12 | bi-allelic |
| C61 | WT | +/+ | 1.0 | heterozygote | +1 | -9 | bi-allelic |
| C62 | mutant | +/+ | 1.0 | heterozygote | +1 | -1 | bi-allelic |
| C63 | mutant | +/+ | 1.0 | heterozygote | -7 | -4 | bi-allelic |
| C64 | WT | +/+ | 1.0 | heterozygote | -1 | -3 | bi-allelic |
| C65 | WT | +/+ | 1.0 | heterozygote | -6 | -3 | bi-allelic |

**Note: The homozygote,bi-allelic and mosaic mutation were defined as only 1 mutation, 2 mutations and more than 2 mutations identified among 20 sanger sequencing, respectively.**

**Table S2. Frequency of mutations induced by the *in vivo* DTM effect resulting in an intended mutant phenotype among the progeny of crosses with its’ wild type genetic background.**

| **T1 line*** | **Recipient× mutator**  **(DTM)** | **Mutant phenotype (n)** | **Population size**  **(n)** | **Mutant phenotype (%)** | **Average** |
| --- | --- | --- | --- | --- | --- |
| CF1-1 | ZC01×CF1-1 | 10 | 29 | 34.48% | 27.98% |
| CF11-15 | ZC01×CF11-15 | 4 | 44 | 9.09% |  |
| CF13-1 | ZC01×CF13-1 | 18 | 83 | 21.69% |  |
| CF18-9 | ZC01×CF18-9 | 6 | 12 | 50.00% |  |
| CF19-1 | ZC01×CF19-1 | 7 | 34 | 20.59% |  |
| CF21-1 | ZC01×CF21-1 | 2 | 5 | 40.00% |  |
| CF22-1 | ZC01×CF22-1 | 8 | 36 | 22.22% |  |
| CF26-5 | ZC01×CF26-5 | 5 | 21 | 23.81% |  |
| CF29-8 | ZC01×CF29-8 | 6 | 29 | 20.69% |  |
| CF30-9 | ZC01×CF30-9 | 25 | 84 | 29.76% |  |
| CF31-8 | ZC01×CF31-8 | 12 | 35 | 34.29% |  |
| CF32-2 | ZC01×CF32-2 | 7 | 24 | 29.17% |  |

Note: * The genetic background of all T1 lines here were ZC01.

**Table S3. Validation of the heritability of mutation induced by DTM effect in the successive F2 generation of 2 F1 crosses.**

| **F1 crosses (recipient line × DTM )** | **F1 individuals** | **lg1 phenotypes of 50 F2 individuals** | **Percentage** |
| --- | --- | --- | --- |
| Huangzao4×CF13-1 | wild type (selfed) | 4/50 | 8.0% |
|  | mutant (selfed) | 49/50 | 98.0% |
| Dan340×CF13-1 | wild type (selfed) | 3/50 | 6.0% |
|  | mutant (selfed) | 49/50 | 98.0% |

**Table S4. The sequence of the mentioned key elements of RNA-guided Cas9 expression cassette in this study.**

| 1. **Sequence of maize ubiquitin promoter with intron used into vector construction** |
| --- |

**> maize ubiquitin promoter with intron**

**1 TCTAGTGCAG TGCAGCGTGA CCCGGTCGTG CCCCTCTCTA GAGATAATGA**

**51 GCATTGCATG TCTAAGTTAT AAAAAATTAC CACATATTTT TTTTGTCACA**

**101 CTTGTTTGAA GTGCAGTTTA TCTATCTTTA TACATATATT TAAACTTTAC**

**151 TCTACGAATA ATATAATCTA TAGTACTACA ATAATATCAG TGTTTTAGAG**

**201 AATCATATAA ATGAACAGTT AGACATGGTC TAAAGGACAA TTGAGTATTT**

**251 TGACAACAGG ACTCTACAGT TTTATCTTTT TAGTGTGCAT GTGTTCTCCT**

**301 TTTTTTTTGC AAATAGCTTC ACCTATATAA TACTTCATCC ATTTTATTAG**

**351 TACATCCATT TAGGGTTTAG GGTTAATGGT TTTTATAGAC TAATTTTTTT**

**401 AGTACATCTA TTTTATTCTA TTTTAGCCTC TAAATTAAGA AAACTAAAAC**

**451 TCTATTTTAG TTTTTTTATT TAATAATTTA GATATAAAAT AGAATAAAAT**

**501 AAAGTGACTA AAAATTAAAC AAATACCCTT TAAGAAATTA AAAAAACTAA**

**551 GGAAACATTT TTCTTGTTTC GAGTAGATAA TGCCAGCCTG TTAAACGCCG**

**601 TCGACGAGTC TAACGGACAC CAACCAGCGA ACCAGCAGCG TCGCGTCGGG**

**651 CCAAGCGAAG CAGACGGCAC GGCATCTCTG TCGCTGCCTC TGGACCCCTC**

**701 TCGAGAGTTC CGCTCCACCG TTGGACTTGC TCCGCTGTCG GCATCCAGAA**

**751 ATTGCGTGGC GGAGCGGCAG ACGTGAGCCG GCACGGCAGG CGGCCTCCTC**

**801 CTCCTCTCAC GGCACGGCAG CTACGGGGGA TTCCTTTCCC ACCGCTCCTT**

**851 CGCTTTCCCT TCCTCGCCCG CCGTAATAAA TAGACACCCC CTCCACACCC**

**901 TCTTTCCCCA ACCTCGTGTT GTTCGGAGCG CACACACACA CAACCAGATC**

**951 TCCCCCAAAT CCACCCGTCG GCACCTCCGC TTCAAGGTAC GCCGCTCGTC**

**1001 CTCCCCCCCC CCCCCTCTCT ACCTTCTCTA GATCGGCGTT CCGGTCCATG**

**1051 GTTAGGGCCC GGTAGTTCTA CTTCTGTTCA TGTTTGTGTT AGATCCGTGT**

**1101 TTGTGTTAGA TCCGTGCTGC TAGCGTTCGT ACACGGATGC GACCTGTACG**

**1151 TCAGACACGT TCTGATTGCT AACTTGCCAG TGTTTCTCTT TGGGGAATCC**

**1201 TGGGATGGCT CTAGCCGTTC CGCAGACGGG ATCGATTTCA TGATTTTTTT**

**1251 TGTTTCGTTG CATAGGGTTT GGTTTGCCCT TTTCCTTTAT TTCAATATAT**

**1301 GCCGTGCACT TGTTTGTCGG GTCATCTTTT CATGCTTTTT TTTGTCTTGG**

**1351 TTGTGATGAT GTGGTCTGGT TGGGCGGTCG TTCTAGATCG GAGTAGAATT**

**1401 CTGTTTCAAA CTACCTGGTG GATTTATTAA TTTTGGATCT GTATGTGTGT**

**1451 GCCATACATA TTCATAGTTA CGAATTGAAG ATGATGGATG GAAATATCGA**

**1501 TCTAGGATAG GTATACATGT TGATGCGGGT TTTACTGATG CATATACAGA**

**1551 GATGCTTTTT GTTCGCTTGG TTGTGATGAT GTGGTGTGGT TGGGCGGTCG**

**1601 TTCATTCGTT CTAGATCGGA GTAGAATACT GTTTCAAACT ACCTGGTGTA**

**1651 TTTATTAATT TTGGAACTGT ATGTGTGTGT CATACATCTT CATAGTTACG**

**1701 AGTTTAAGAT GGATGGAAAT ATCGATCTAG GATAGGTATA CATGTTGATG**

**1751 TGGGTTTTAC TGATGCATAT ACATGATGGC ATATGCAGCA TCTATTCATA**

**1801 TGCTCTAACC TTGAGTACCT ATCTATTATA ATAAACAAGT ATGTTTTATA**

**1851 ATTATTTTGA TCTTGATATA CTTGGATGAT GGCATATGCA GCAGCTATAT**

**1901 GTGGATTTTT TTAGCCCTGC CTTCATACGC TATTTATTTG CTTGGTACTG**

**1951 TTTCTTTTGT CGATGCTCAC CCTGTTGTTT GGTGTTACTT CTGCACT**

| 1. **Sequence of the coding sequence of SpCas9 sequence used into vector construction** |
| --- |

**> The coding sequence of SpCas9 sequence**

**1 ATGGACTATA AGGACCACGA CGGAGACTAC AAGGATCATG ATATTGATTA**

**51 CAAAGACGAT GACGATAAGA TGGCCCCAAA GAAGAAGCGG AAGGTCGGTA**

**101 TCCACGGAGT CCCAGCAGCC GACAAGAAGT ACAGCATCGG CCTGGACATC**

**151 GGCACCAACT CTGTGGGCTG GGCCGTGATC ACCGACGAGT ACAAGGTGCC**

**201 CAGCAAGAAA TTCAAGGTGC TGGGCAACAC CGACCGGCAC AGCATCAAGA**

**251 AGAACCTGAT CGGAGCCCTG CTGTTCGACA GCGGCGAAAC AGCCGAGGCC**

**301 ACCCGGCTGA AGAGAACCGC CAGAAGAAGA TACACCAGAC GGAAGAACCG**

**351 GATCTGCTAT CTGCAAGAGA TCTTCAGCAA CGAGATGGCC AAGGTGGACG**

**401 ACAGCTTCTT CCACAGACTG GAAGAGTCCT TCCTGGTGGA AGAGGATAAG**

**451 AAGCACGAGC GGCACCCCAT CTTCGGCAAC ATCGTGGACG AGGTGGCCTA**

**501 CCACGAGAAG TACCCCACCA TCTACCACCT GAGAAAGAAA CTGGTGGACA**

**551 GCACCGACAA GGCCGACCTG CGGCTGATCT ATCTGGCCCT GGCCCACATG**

**601 ATCAAGTTCC GGGGCCACTT CCTGATCGAG GGCGACCTGA ACCCCGACAA**

**651 CAGCGACGTG GACAAGCTGT TCATCCAGCT GGTGCAGACC TACAACCAGC**

**701 TGTTCGAGGA AAACCCCATC AACGCCAGCG GCGTGGACGC CAAGGCCATC**

**751 CTGTCTGCCA GACTGAGCAA GAGCAGACGG CTGGAAAATC TGATCGCCCA**

**801 GCTGCCCGGC GAGAAGAAGA ATGGCCTGTT CGGAAACCTG ATTGCCCTGA**

**851 GCCTGGGCCT GACCCCCAAC TTCAAGAGCA ACTTCGACCT GGCCGAGGAT**

**901 GCCAAACTGC AGCTGAGCAA GGACACCTAC GACGACGACC TGGACAACCT**

**951 GCTGGCCCAG ATCGGCGACC AGTACGCCGA CCTGTTTCTG GCCGCCAAGA**

**1001 ACCTGTCCGA CGCCATCCTG CTGAGCGACA TCCTGAGAGT GAACACCGAG**

**1051 ATCACCAAGG CCCCCCTGAG CGCCTCTATG ATCAAGAGAT ACGACGAGCA**

**1101 CCACCAGGAC CTGACCCTGC TGAAAGCTCT CGTGCGGCAG CAGCTGCCTG**

**1151 AGAAGTACAA AGAGATTTTC TTCGACCAGA GCAAGAACGG CTACGCCGGC**

**1201 TACATTGACG GCGGAGCCAG CCAGGAAGAG TTCTACAAGT TCATCAAGCC**

**1251 CATCCTGGAA AAGATGGACG GCACCGAGGA ACTGCTCGTG AAGCTGAACA**

**1301 GAGAGGACCT GCTGCGGAAG CAGCGGACCT TCGACAACGG CAGCATCCCC**

**1351 CACCAGATCC ACCTGGGAGA GCTGCACGCC ATTCTGCGGC GGCAGGAAGA**

**1401 TTTTTACCCA TTCCTGAAGG ACAACCGGGA AAAGATCGAG AAGATCCTGA**

**1451 CCTTCCGCAT CCCCTACTAC GTGGGCCCTC TGGCCAGGGG AAACAGCAGA**

**1501 TTCGCCTGGA TGACCAGAAA GAGCGAGGAA ACCATCACCC CCTGGAACTT**

**1551 CGAGGAAGTG GTGGACAAGG GCGCTTCCGC CCAGAGCTTC ATCGAGCGGA**

**1601 TGACCAACTT CGATAAGAAC CTGCCCAACG AGAAGGTGCT GCCCAAGCAC**

**1651 AGCCTGCTGT ACGAGTACTT CACCGTGTAT AACGAGCTGA CCAAAGTGAA**

**1701 ATACGTGACC GAGGGAATGA GAAAGCCCGC CTTCCTGAGC GGCGAGCAGA**

**1751 AAAAGGCCAT CGTGGACCTG CTGTTCAAGA CCAACCGGAA AGTGACCGTG**

**1801 AAGCAGCTGA AAGAGGACTA CTTCAAGAAA ATCGAGTGCT TCGACTCCGT**

**1851 GGAAATCTCC GGCGTGGAAG ATCGGTTCAA CGCCTCCCTG GGCACATACC**

**1901 ACGATCTGCT GAAAATTATC AAGGACAAGG ACTTCCTGGA CAATGAGGAA**

**1951 AACGAGGACA TTCTGGAAGA TATCGTGCTG ACCCTGACAC TGTTTGAGGA**

**2001 CAGAGAGATG ATCGAGGAAC GGCTGAAAAC CTATGCCCAC CTGTTCGACG**

**2051 ACAAAGTGAT GAAGCAGCTG AAGCGGCGGA GATACACCGG CTGGGGCAGG**

**2101 CTGAGCCGGA AGCTGATCAA CGGCATCCGG GACAAGCAGT CCGGCAAGAC**

**2151 AATCCTGGAT TTCCTGAAGT CCGACGGCTT CGCCAACAGA AACTTCATGC**

**2201 AGCTGATCCA CGACGACAGC CTGACCTTTA AAGAGGACAT CCAGAAAGCC**

**2251 CAGGTGTCCG GCCAGGGCGA TAGCCTGCAC GAGCACATTG CCAATCTGGC**

**2301 CGGCAGCCCC GCCATTAAGA AGGGCATCCT GCAGACAGTG AAGGTGGTGG**

**2351 ACGAGCTCGT GAAAGTGATG GGCCGGCACA AGCCCGAGAA CATCGTGATC**

**2401 GAAATGGCCA GAGAGAACCA GACCACCCAG AAGGGACAGA AGAACAGCCG**

**2451 CGAGAGAATG AAGCGGATCG AAGAGGGCAT CAAAGAGCTG GGCAGCCAGA**

**2501 TCCTGAAAGA ACACCCCGTG GAAAACACCC AGCTGCAGAA CGAGAAGCTG**

**2551 TACCTGTACT ACCTGCAGAA TGGGCGGGAT ATGTACGTGG ACCAGGAACT**

**2601 GGACATCAAC CGGCTGTCCG ACTACGATGT GGACCATATC GTGCCTCAGA**

**2651 GCTTTCTGAA GGACGACTCC ATCGACAACA AGGTGCTGAC CAGAAGCGAC**

**2701 AAGAACCGGG GCAAGAGCGA CAACGTGCCC TCCGAAGAGG TCGTGAAGAA**

**2751 GATGAAGAAC TACTGGCGGC AGCTGCTGAA CGCCAAGCTG ATTACCCAGA**

**2801 GAAAGTTCGA CAATCTGACC AAGGCCGAGA GAGGCGGCCT GAGCGAACTG**

**2851 GATAAGGCCG GCTTCATCAA GAGACAGCTG GTGGAAACCC GGCAGATCAC**

**2901 AAAGCACGTG GCACAGATCC TGGACTCCCG GATGAACACT AAGTACGACG**

**2951 AGAATGACAA GCTGATCCGG GAAGTGAAAG TGATCACCCT GAAGTCCAAG**

**3001 CTGGTGTCCG ATTTCCGGAA GGATTTCCAG TTTTACAAAG TGCGCGAGAT**

**3051 CAACAACTAC CACCACGCCC ACGACGCCTA CCTGAACGCC GTCGTGGGAA**

**3101 CCGCCCTGAT CAAAAAGTAC CCTAAGCTGG AAAGCGAGTT CGTGTACGGC**

**3151 GACTACAAGG TGTACGACGT GCGGAAGATG ATCGCCAAGA GCGAGCAGGA**

**3201 AATCGGCAAG GCTACCGCCA AGTACTTCTT CTACAGCAAC ATCATGAACT**

**3251 TTTTCAAGAC CGAGATTACC CTGGCCAACG GCGAGATCCG GAAGCGGCCT**

**3301 CTGATCGAGA CAAACGGCGA AACCGGGGAG ATCGTGTGGG ATAAGGGCCG**

**3351 GGATTTTGCC ACCGTGCGGA AAGTGCTGAG CATGCCCCAA GTGAATATCG**

**3401 TGAAAAAGAC CGAGGTGCAG ACAGGCGGCT TCAGCAAAGA GTCTATCCTG**

**3451 CCCAAGAGGA ACAGCGATAA GCTGATCGCC AGAAAGAAGG ACTGGGACCC**

**3501 TAAGAAGTAC GGCGGCTTCG ACAGCCCCAC CGTGGCCTAT TCTGTGCTGG**

**3551 TGGTGGCCAA AGTGGAAAAG GGCAAGTCCA AGAAACTGAA GAGTGTGAAA**

**3601 GAGCTGCTGG GGATCACCAT CATGGAAAGA AGCAGCTTCG AGAAGAATCC**

**3651 CATCGACTTT CTGGAAGCCA AGGGCTACAA AGAAGTGAAA AAGGACCTGA**

**3701 TCATCAAGCT GCCTAAGTAC TCCCTGTTCG AGCTGGAAAA CGGCCGGAAG**

**3751 AGAATGCTGG CCTCTGCCGG CGAACTGCAG AAGGGAAACG AACTGGCCCT**

**3801 GCCCTCCAAA TATGTGAACT TCCTGTACCT GGCCAGCCAC TATGAGAAGC**

**3851 TGAAGGGCTC CCCCGAGGAT AATGAGCAGA AACAGCTGTT TGTGGAACAG**

**3901 CACAAGCACT ACCTGGACGA GATCATCGAG CAGATCAGCG AGTTCTCCAA**

**3951 GAGAGTGATC CTGGCCGACG CTAATCTGGA CAAAGTGCTG TCCGCCTACA**

**4001 ACAAGCACCG GGATAAGCCC ATCAGAGAGC AGGCCGAGAA TATCATCCAC**

**4051 CTGTTTACCC TGACCAATCT GGGAGCCCCT GCCGCCTTCA AGTACTTTGA**

**4101 CACCACCATC GACCGGAAGA GGTACACCAG CACCAAAGAG GTGCTGGACG**

**4151 CCACCCTGAT CCACCAGAGC ATCACCGGCC TGTACGAGAC ACGGATCGAC**

**4201 CTGTCTCAGC TGGGAGGCGA CAAAAGGCCG GCGGCCACGA AAAAGGCCGG**

**4251 CCAGGCAAAA AAGAAAAAGT AA**

| 1. **Sequence of SV40 NLS used into vector construction** |
| --- |

**> SV40 NLS sequence**

**1 ATGGCCCCAA AGAAGAAGCG GAAGGTCGGT ATCCACGGAG TCCCAGCAGC**

**51 C**

| 1. **Sequence of Nucleoplasmin NLS used into vector construction** |
| --- |

**> Nucleoplasmin NLS sequence**

**1 AAAAGGCCGG CGGCCACGAA AAAGGCCGGC CAGGCAAAAA AGAAAAAG**

| 1. **Sequence of *ZmU6-6* PolIII promoter used into vector construction** |
| --- |

**>ZmU6-6 PolIII promoter sequence**

**1 AACCTCGCTT GTATAGTTCC TTGTGCTCTA ACACACGATG ATGATAAGTC**

**51 GTAAAATAGT GGTGTCCAAA GAATTTCCAG GCCCAGTTGT AAAAGCTAAA**

**101 ATGCTATTCG AATTTCTACT AGCAGTAAGT CGTGTTTAGA AATTATTTTT**

**151 TTATATACCT TTTTTCCTTC TATGTACAGT AGGACACAGT GTCAGCGCCG**

**201 CGTTGACGGA GAATATTTGC AAAAAAGTAA AAGAGAAAGT CATAGCGGCG**

**251 TATGTGCCAA AAACTTCGTC ACAGAGAGGG CCATAAGAAA CATGGCCCAC**

**301 GGCCCAATAC GAAGCACCGC GACGAAGCCC AAACAGCAGT CCGTAGGTGG**

**351 AGCAAAGCGC TGGGTAATAC GCAAACGTTT TGTCCCACCT TGACTAATCA**

**401 CAAGAGTGGA GCGTACCTTA TAAACCGAGC CGCAAGCACC GAATT**

| 1. **Sequence of gRNA scaffold sequence used into vector construction** |
| --- |
| **>gRNA scaffold sequence （Universal sgRNA sequence）**  **1 GTTTTAGAGC TAGAAATAGC AAGTTAAAAT AAGGCTAGTC CGTTATCAAC**  **51 TTGAAAAAGT GGCACCGAGT CGGTGCTTTT TTT** |
| 1. **Sequence of sgRNA targeting sequence and flanking region of *ZmLG1* locus in ZC01,the transformation recipient and DTM donor.**   **>ZC01 ZmLG1 sgRNA targeting region**  **1 ATCAACTGCT GGGCTACAAC CTCGAGGCCA ACTCGCTGGC CCTCCTGCCC**  **51 CCGTCCAACG CCGCCGCCGC TCACCACCAC ACCACCTTCG CCGGCGGCCA**  **101 CAGCCCCCAC GACATCCTCC ACTTCTACAC ACCTCCTCCT TCCGCCGCCT**  **151 CGCACTACCT CGCCGCCGCC GCCGGCAACC CCTACAGCCA CTTAGTCTCC**  **201 GCGCCCGGGA CCACCTTCCA CCAGACCTCG TCGTCCTACT ACCCGCCGGC**  **251 GGCGGCGGCG CAGGCCGCGC CCGAGTACTA CTTCCCCACC CTCGTCAGCT**  **301 CCGCCGAGGA GAACATGGCC AGCTTCGCCG CCACGCAGCT CGGCCTCAAC**  **351 CTCGGCTACC GCACCTACTT CCCCCCCAGA GGAGGCTACA CGTAC**  **Note: PAM sequence; sgRNA targeting region** |
